# Fiber Optic Projection-Imaging System for Shape Measurement in Confined Space

**DOI:** 10.1155/2014/206569

**Published:** 2014-02-10

**Authors:** Lujie Chen, Viswanath Bavigadda, Theodoros Kofidis, Robert D. Howe

**Affiliations:** ^1^Engineering Product Design, Singapore University of Technology and Design, Singapore 138682; ^2^Yong Loo Lin School of Medicine, National University of Singapore, Singapore 119228; ^3^School of Engineering and Applied Sciences, Harvard University, Cambridge, MA 02138, USA

## Abstract

A fiber-based projection-imaging system is proposed for shape measurement in confined space. Owing to the flexibility of imaging fibers, the system can be used in special scenarios that are difficult for conventional experimental setups. Three experiments: open space, closed space, and underwater are designed to demonstrate the strength and weakness of the system. It is shown that when proper alignment is possible, relatively high accuracy can be achieved; the error is less than 2% of the overall height of a specimen. In situations where alignment is difficult, significantly increased error is observed. The error is in the form of gross-scale geometrical distortion; for example, flat surface is reconstructed with curvature. In addition, the imaging fibers may introduce fine-scale noise into phase measurement, which has to be suppressed by smoothing filters. Based on results and analysis, it is found that although a fiber-based system has its unique strength, existing calibration and processing methods for fringe patterns have to be modified to overcome its drawbacks so as to accommodate wider applications.

## 1. Introduction

Substantial research and development efforts have gone into enhancing the accuracy, functionality, and implementation of various optical techniques based on the principle of holography, interferometry, morié, structured light projection, stereo vision, and photometrology. Optical systems for three-dimensional (3D) shape measurement have found wide range of applications. They encompass metrological evaluation in macro-, meso-, and microscales.

Existing approaches, commercial systems, and well-known experimental setups have standardized the solution to many different measurement tasks in diverse scenarios. At macroscale, stereo vision and photometric methods are commonly used to reconstruct 3D shape of objects whose dimensions are above one cubic meter [1]. At mesoscale, desktop-sized objects can be conveniently measured on an optical table in a lab environment. Depending on the objective of evaluation (shape or displacement), a technique may be chosen that best suits the problem under consideration. Holography [2] and interferometry [3] are suited for displacement measurement; morié [4] and structured light projection [5] are suited for shape measurement. There are certainly variations, for example, white light interferometry for shape measurement [6]. Some method, such as digital image correlation [7], can even achieve both in one go. At microscale, precise optical alignment of a system becomes increasingly important. Most implementations of optical techniques are based on a microscope to take advantage of its well designed lens relay [8].

While the main stream of optical system development assumes that specimen can be put at a designated position, this is not possible in *in-situ* measurement; thus developers have been motivated to incorporate optical techniques in special devices, such as endoscopes and fiber scopes. Over the years, holograms were produced out of fiber optic systems [9–11]; morié fringe patterns were generated through single-mode fibers, with potential use in medical diagnosis [12]; fringe projection was achieved by transmitting the image of a grating through fibers to make measurement on microobjects [13]. Data-processing methods: phase-shifting and Fourier transform were applied to fringe patterns obtained by an endoscope or a fiber scope, just as in free-space scenarios [14–16]. Photometric approaches were also adapted to these devices [17, 18].

Several trends along the development of endoscopic shape measurement have been observed. First, more digital components are integrated in a system; for example, gratings were replaced by digital mirror devices (DMD) or spatial light modulation (SLM) units [8, 19]. Second, special projection patterns were applied to retrieve 3D information [20–22]. Third, the applications were more specific and the solutions were more customized [23–25]. Last but not least, low-cost, off-the-shelf digital projectors were demonstrated to be a feasible projection unit [26, 27]. They provide similar level of flexibility to the expensive DMD or SLM devices in generating projection patterns.

However, as an off-the-shelf digital projector has its own built-in optics, not optimized for microscopic applications, severe optical distortion is likely to occur. In this paper, we describe a fiber optic projection-imaging system for shape measurement; compare the measurement results of a MEMS component in three scenarios: free space, confined space and underwater; discuss the pros and cons of the system; and suggest tentative approaches to enhance the performance of the low-cost digital-projector-based fiberscope as a high-precision measurement tool.

## 2. Principle

**Figure 1:**
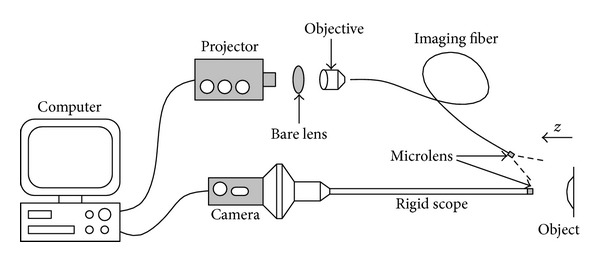
System diagram.

Constructing a structured light projection system based on fiber optics is straight forward in principle, as illustrated in Figure 1. The major difference from a nonfiber version lies in the use of imaging fiber bundles for directing the light, either in projection or in imaging, or both. Note that, despite its name, the imaging fiber works equally well for transmitting the projected light as for collecting light to pass to a camera. Depending on requirement, flexible or rigid imaging fibers may be incorporated. On the market, there are commercial rigid scopes that work with various types of cameras; hence, the real challenge in optical alignment is to couple the light from the projector to the imaging fiber.

Two components: a bare lens and an objective lens, as indicated in Figure 1, are necessary for light coupling. Usually, an off-the-shelf digital projector has a big divergence angle. The bare lens is used to reduce the size of the projected optical cone so that a large portion of the projection area can enter the objective lens. Without the bare lens, most pixels of the projector will be wasted and the subsequent resolution will be low. Rule of thumb for choosing a suitable bare lens is to get at least as many pixels as the resolution of the imaging fiber into the objective lens. At the distal end of the fiber, a microlens is often attached to increase the numerical aperture (NA) of the imaging fiber. Consequently, a fairly complicated lens relay is in between the projector chip and the pattern projected on an object. The relay consists of the projects' built-in lens, the bare lens, the objective lens, and the microlens. Misalignment of the optical center of these lenses will cause distortion. Same applies to the imaging optical path.

The following procedures are applied to system calibration. They have been proven to be valid and accurate for nonfiber based fringe projection systems [28, 29]. A pinhole model is assumed on both the projection and the camera optics.

To calibrate the camera optics, a chessboard pattern is imaged at two positions along the *z* direction (Figure 1), with a known shift in between. The actual size of the squares on the chessboard is known too; hence, the 3D coordinate of all corners of the pattern is known. The origin of the world coordinate may be chosen arbitrarily. For instance, the origin may be the first chessboard corner on the far *z* direction position. The corresponding corner points on the images can be extracted at subpixel accuracy. Then, a matrix that represents the mapping between the image (2D) and the world (3D) points is calculated based on numerical methods described in [1]:

(1) $$\begin{array}{c} {\left(x_{i},y_{i},1\right)}^{T}=M\cdot {\left(x_{w},y_{w},z_{w},1\right)}^{T}, \end{array}$$

where *M* is the camera matrix, *T* denotes matrix transpose, *x*_*i*_ and *y*_*i*_ are coordinates of an imaged corner point, *x*_*w*_, *y*_*w*_, and *z*_*w*_ are coordinates of the corresponding world point. *M* is used later to retrieve 3D coordinates of an object surface.

**Figure 2:**
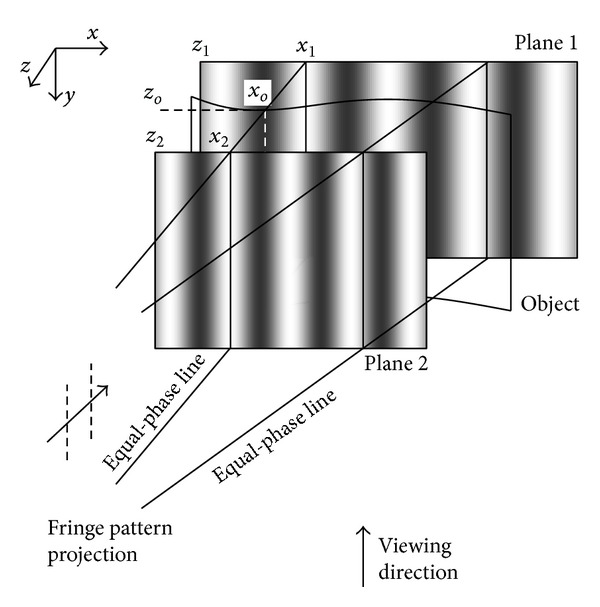
Calibration of phase-to-height relationship.

To calibrate the projection optics, the matrix of the lens relay is not calculated but the phase-to-height relationship is inferred from phase maps at two positions along *z* direction [28, 29]. As shown in Figure 2, planes 1 and 2 are two reference planes with a pure *z* direction shift. “Object” indicates an object surface in between the reference planes. At (*x*_1_, *z*_1_), (*x*_2_, *z*_2_) and (*x*_*o*_, *z*_*o*_), the fringe patterns should have identical phase values, which can be found by phase mapping [30]. On each equal-phase line, three *x* coordinates, *x*_1_, *x*_2_, and *x*_*o*_, should be mapped to subpixel accuracy; hence, the unknown object surface height *z*_*o*_ can be calculated by

(2) $$\begin{array}{c} z_{o}=\frac{x_{o}-x_{1}}{x_{2}-x_{1}}z_{2}, \end{array}$$

assuming *z*_1_ = 0. Note that (2) is theoretically valid even if an object surface point is beyond the reference planes.

After phase-to-height conversion, an object point obtains a 3D coordinate of mixed units. The *x* and *y* coordinates of the point are in the image coordinate, with a unit of pixel. *z* is a world coordinate, with a unit of an actual distance, such as millimeter. To obtain *x* and *y* in the world coordinate, (1) should be used again. Since *M* is known after calibration of the camera optics, there are only two unknowns *x*_*w*_ and *y*_*w*_. The matrix representation contains three equations, two of which are linearly independent; hence, *x*_*w*_ and *y*_*w*_ can be solved. This completes the measurement process and the resultant object surface is in the 3D world coordinate.

The calibration is a relatively time-consuming procedure and is expected to be performed only when the system geometrical configuration is modified.

## 3. Experiment

**Figure 3:**
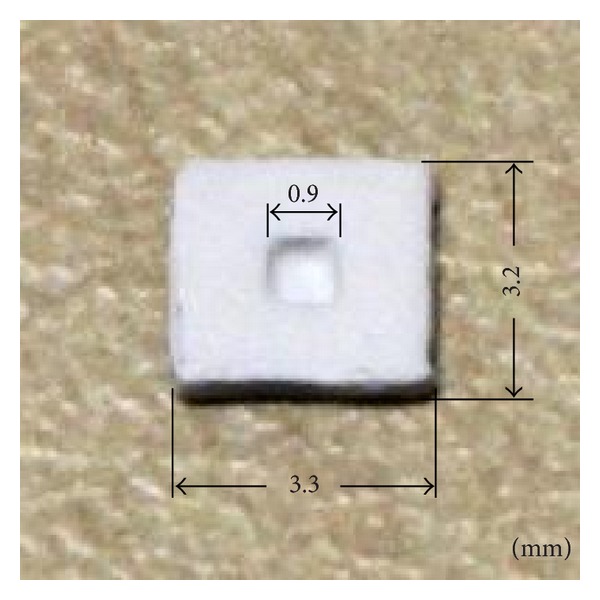
A MEMS component with a thickness of 0.55 mm. The surface was treated with diffusive paint.

Three experiments were conducted on a MEMS component shown in Figure 3. They represent different *in-situ* scenarios: open space, closed space and underwater. 3D measurement in closed space, and underwater is challenging, where a fiber-based projection-imaging system finds its special application.

**Figure 4:**
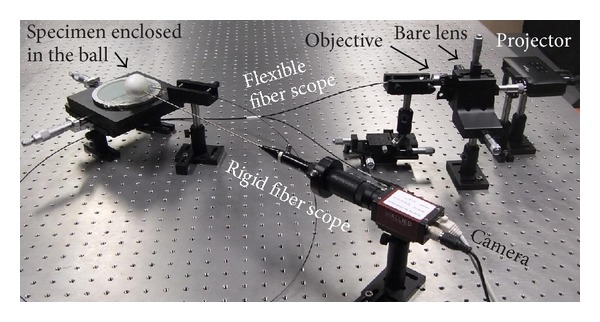
Experimental setup of the closed-space scenario.

Figure 4 shows the experimental setup of the closed-space scenario; the specimen was enclosed in a ping pong ball. The imaging optical path consisted of a CCD camera (Allied Vision Technology, Manta G-504B mono) and a rigid fiber scope. The projection path consisted of a miniprojector, a bare lens (attached to and behind the 3D stage), an objective lens and a flexible fiber scope (Fujikura FIGH-15-600N). Both the rigid and the flexible fiber scopes had an integrated microlens, with a divergent angle of around 100 degrees. The experimental setup of the other two scenarios was similar.

The flexible fiber bundle (Fujikura FIGH-15-600N) has 15,000 pixels with a working distance of 5 mm. It has an outer diameter of 1.3 mm. Specification of the rigid fiber scope is not on record unfortunately but based on our tests, it has 10,000 to 15,000 pixels with a working distance of 4 mm. It has an outer diameter of 2 mm. An adjustable lens at the camera end of the scope is quite useful in getting relatively good focus; hence, defocus is not present as a big challenge. Like any fringe projection system based on triangulation, projection shadow will cause trouble in data processing. However, due to confined space, the angle between the two fiber scopes is small: 5–10 degrees; there is little projection shadow caused by the object surface variation.

**Figure 5:**
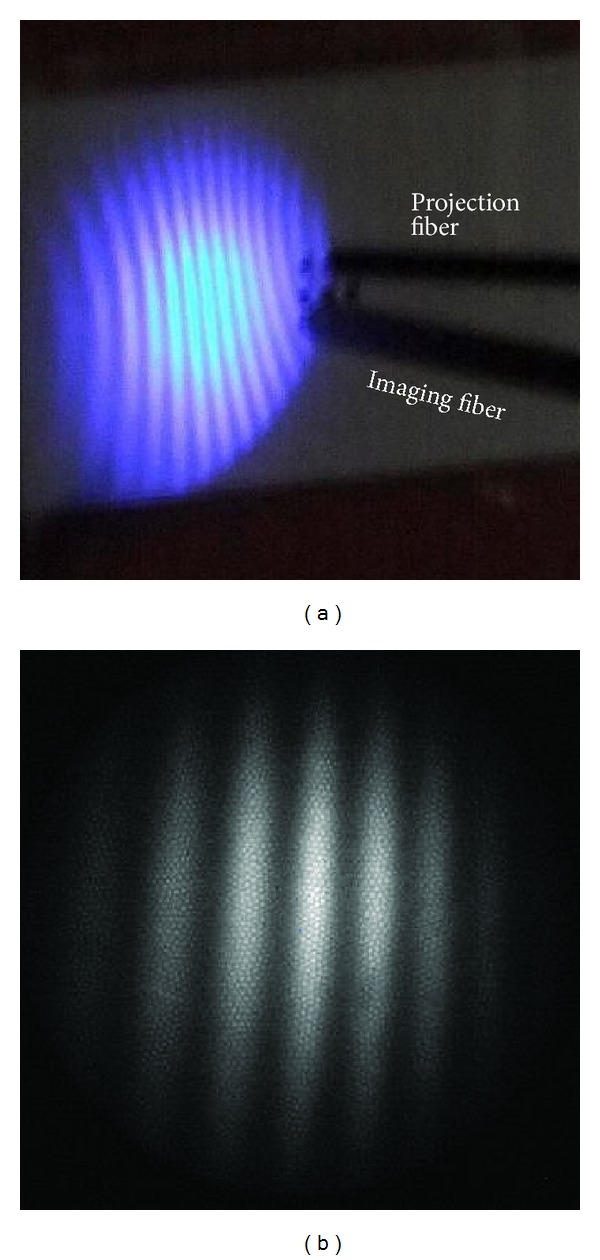
A fringe pattern projected on a reference plane, as seen (a) by an observer and (b) by the camera attached to the rigid fiber scope.

**Figure 6:**
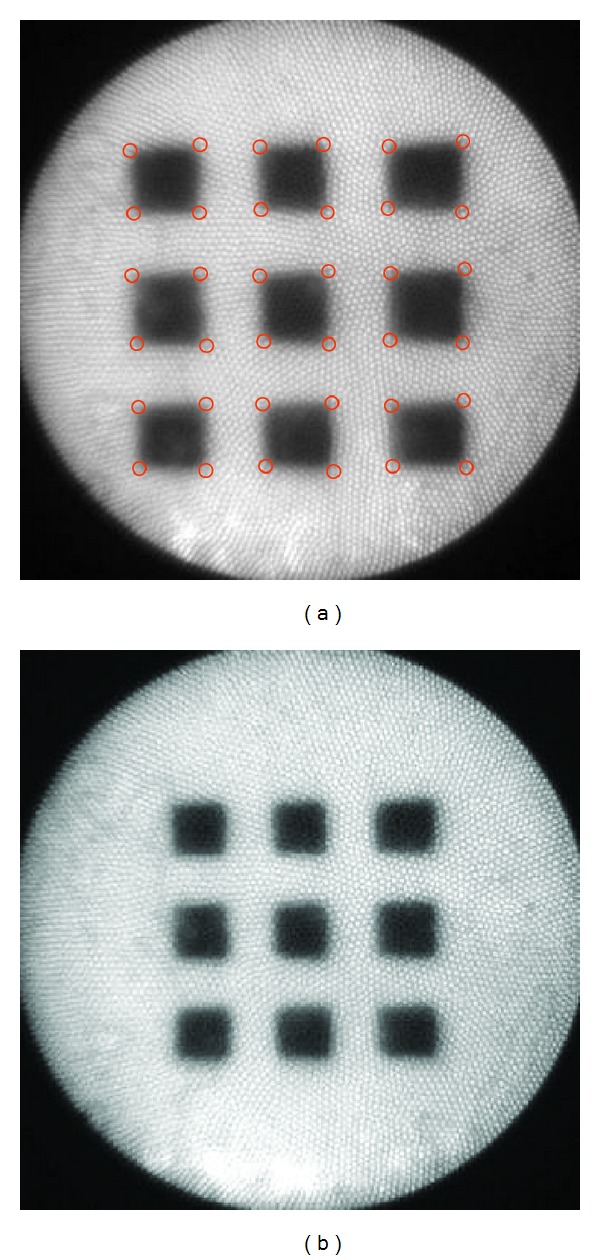
A chessboard pattern for camera calibration, recorded at (a) near and (b) far *z* positions. Red circles in (a) indicate the automatically detected corner points.

Figure 5 shows a typical fringe pattern projected on a reference plane. Relatively large radial distortion can be seen by an observer in Figure 5(a); nevertheless, the pattern recorded by the CCD camera, Figure 5(b), exhibits less radial distortion because (1) it was the central portion of the projected area and (2) the imaging optics had radial distortion too, which happened to cancel out that of the projection optics. In all experiments, the *z* direction shift of the reference plane was introduced through a mechanical micrometer.  Figure 6 shows the chessboard pattern for camera calibration, recorded at two *z* positions. They were on the same positions as where the reference fringe patterns were captured.

**Figure 7:**
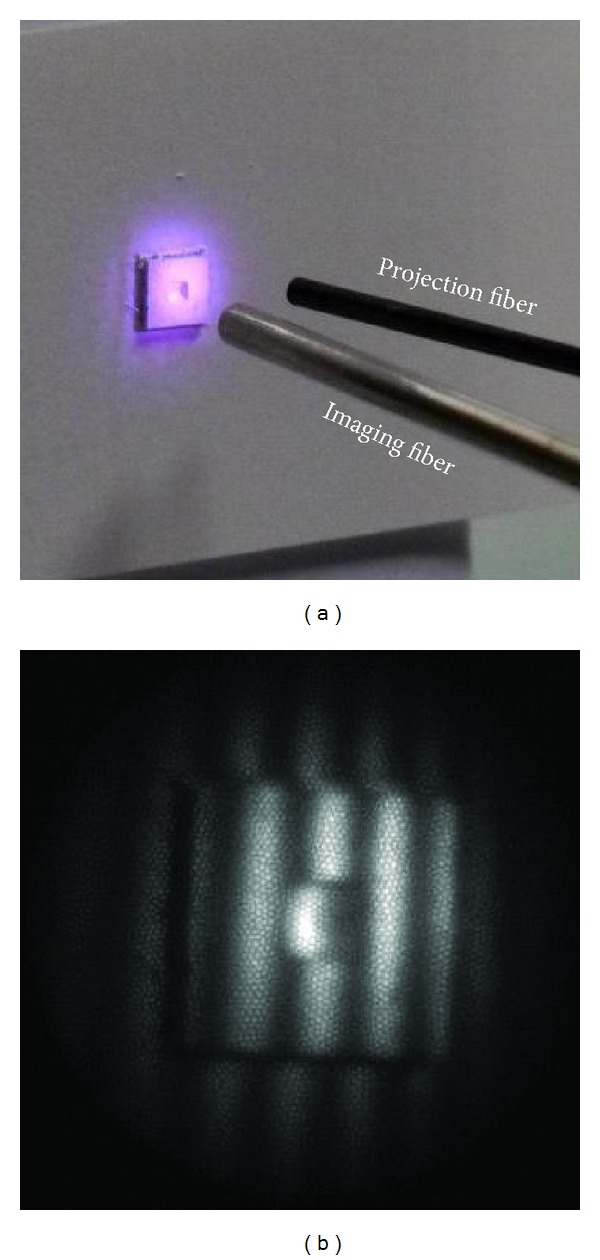
Open-space scenario. (a) Closeup of the fiber distal end. (b) A fringe pattern recorded by the camera.

In the open-space experiment, Figure 7(a), the specimen was aligned perpendicular to the viewing direction, ideal for 3D measurement.  Figure 7(b) shows a typical fringe pattern obtained. Result of this experiment is an indication of the best-scenario case achievable by the system, since there is no constraint in space.

**Figure 8:**
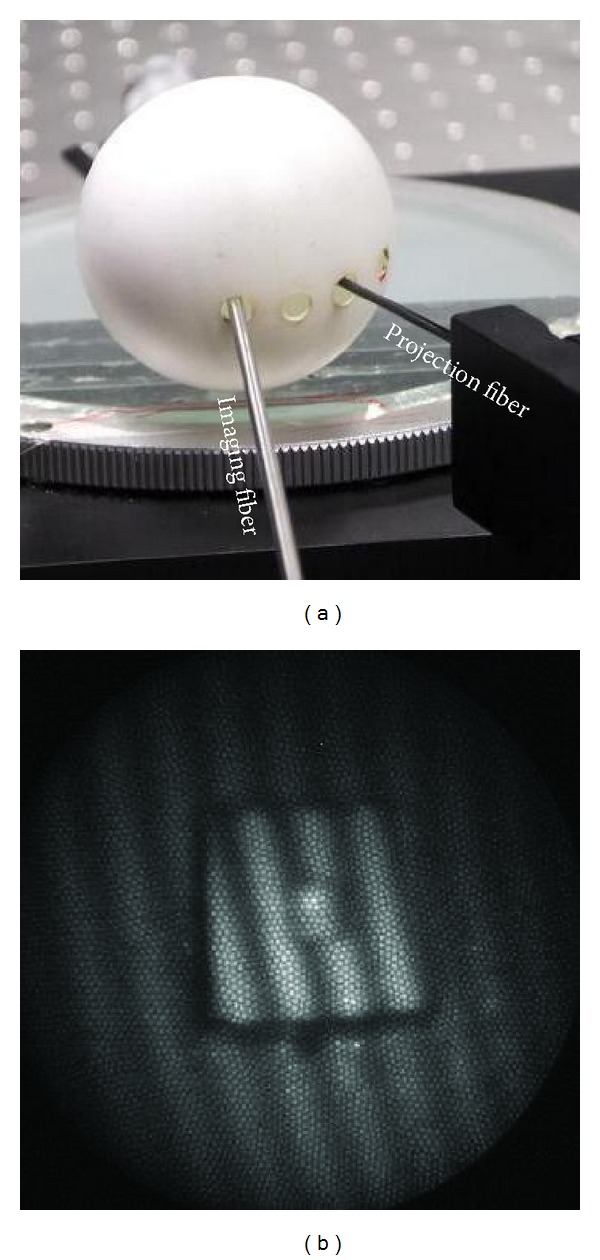
Closed-space scenario. (a) Several holes were created on the surface of the ping pong to provide access for the fibers. (b) A fringe pattern recorded by the camera. Zooming in the picture, one can see the image of lots of individual fiber ends.

In the closed-space experiment, the specimen was attached to the inner surface of a ping pong. Several holes were created on the ping pong to provide access for the fibers, as shown in Figure 8(a).  Figure 8(b) shows a typical fringe pattern obtained, in which one can see lots of individual fiber ends of the imaging fiber bundle. If they are in focus as in this picture, it implies that the imaging optics is in focus as well. In the closed-space scenario, alignment of the specimen with the projection or imaging optics is difficult. The subsequent side-effect will be discussed in Section 4.

**Figure 9:**
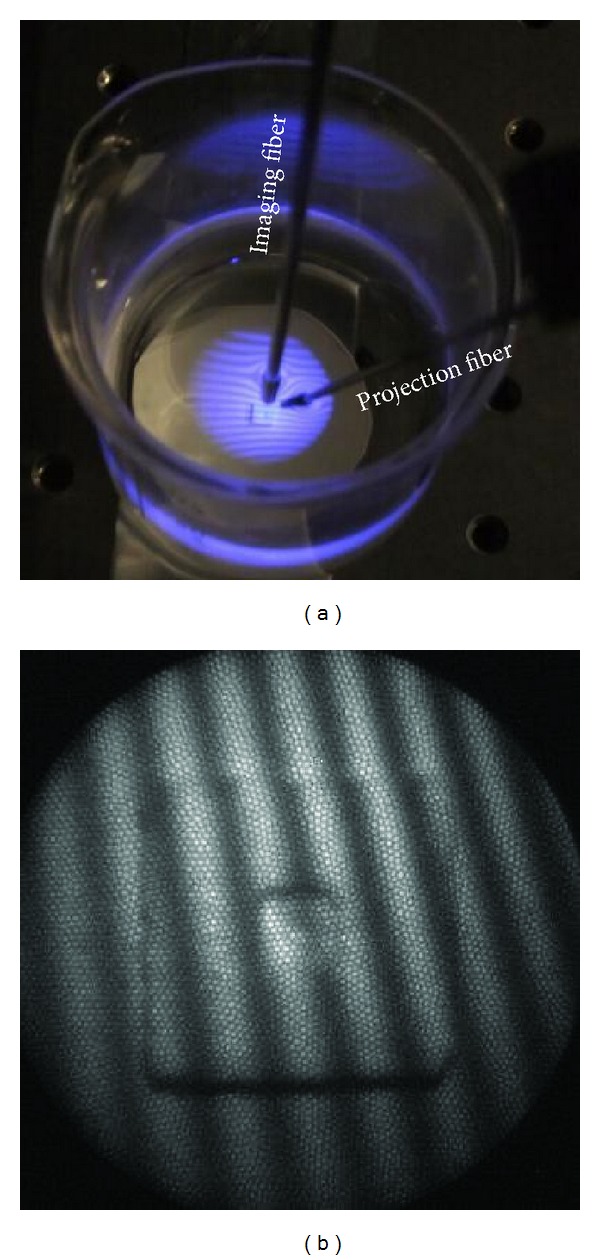
Underwater scenario. Both fibers were dipped in the water. A fringe pattern as seen (a) by an observer and (b) by the camera attached to the rigid fiber scope.

The third experiment was aimed at testing the system for underwater measurement. There were two major challenges: first, the working space was confined and, second, projection or imaging through both air and water is in general not feasible because of refraction at the air-water interface. A fiber-based system is a good candidate for such *in-situ* measurement tasks. As shown in Figure 9(a), the fibers were dipped in the water. The fringes around the fibers, as seen by an observer, were indeed distorted due to refraction; however, those seen by the camera (Figure 9(b)) were not because the reflected light was collected by the imaging fiber in the water without passing through the air-water interface.

## 4. Results and Discussion

**Figure 10:**
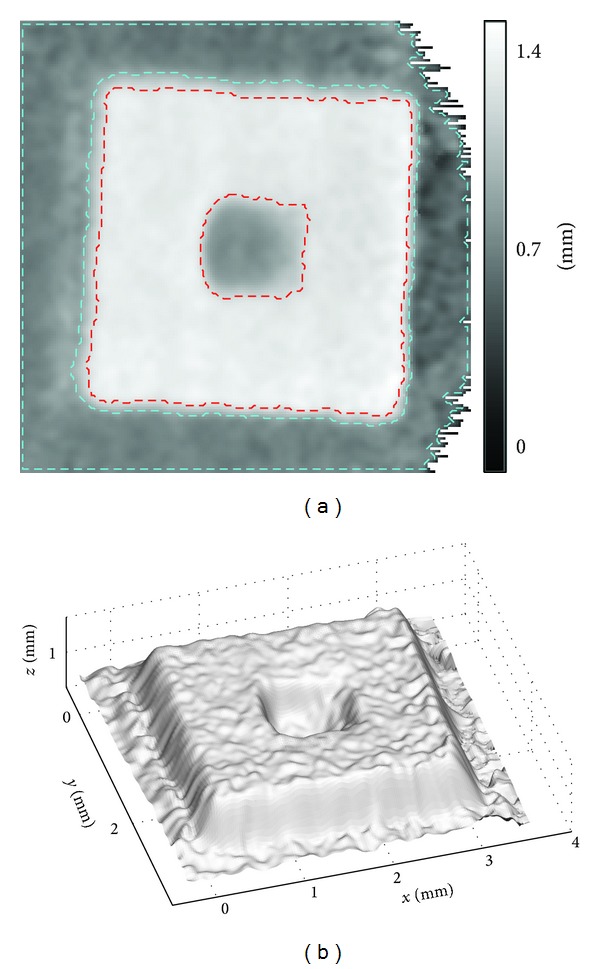
Open-space experiment. (a) Surface height map in 2D and (b) surface profile in 3D of the MEMS component.

Figure 10 shows the surface profile of the MEMS component obtained in the open-space experiment; (a) is the height map visualized in 2D, where *x* and *y* are in pixel unit and the intensity indicates the height in millimeter; in (b), all coordinates are converted to the actual distance based on (1), as described in Section 2. The right-top and right-bottom corners of Figure 10(a) contain invalid phase mapping data due to limited field of view in the reference and the object phase maps. They are masked in white. The immediate phase mapping results are very noisy; hence, 3-by-3 median followed by mean filtering is applied to suppress the noise. The images shown are results after smoothing. The noise is mostly caused by void regions in a fiber bundle: this issue will be further discussed in the underwater experiment.

The specimen has a thickness of 0.55 mm, measured by a calliper. The value is used as a reference to evaluate the overall measurement accuracy. In Figure 10(a), the regions enclosed by red dashed lines are the front surface of the specimen and that enclosed by the cyan dashed lines are the base plane. The average height difference between these two regions is 0.56 mm, obtained by the optical method, which is in good agreement with that obtained by the calliper. The depth of the central dip is not known. Based on the optical measurement, it is around 0.46 mm from the front surface. Visual inspection from different viewing angles suggests that there is no obvious measurement error.

**Figure 11:**
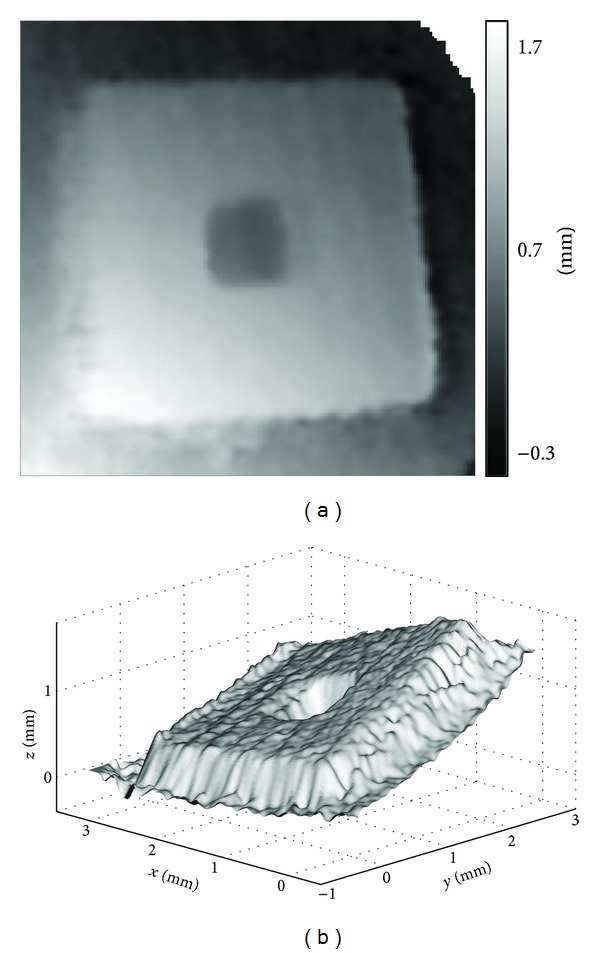
Closed-space experiment. (a) Surface height map in 2D and (b) surface profile in 3D of the MEMS component.

Figure 11 shows in 2D and 3D, respectively, the surface profile of the specimen obtained in the closed-space experiment. As can be seen, the surface is tilted, indicating that the specimen was not perpendicular to the imaging fiber, nor to the reference plane, during the experiment. This is not surprising, since the MEMS component was enclosed in a ping pong (Figure 8(a)) and alignment was difficult. Such imperfect alignment is typical in closed-space scenarios, where accessibility is limited. The results reveal a problem of distortion: the flat front surface and the base plane become curved.

**Figure 12:**
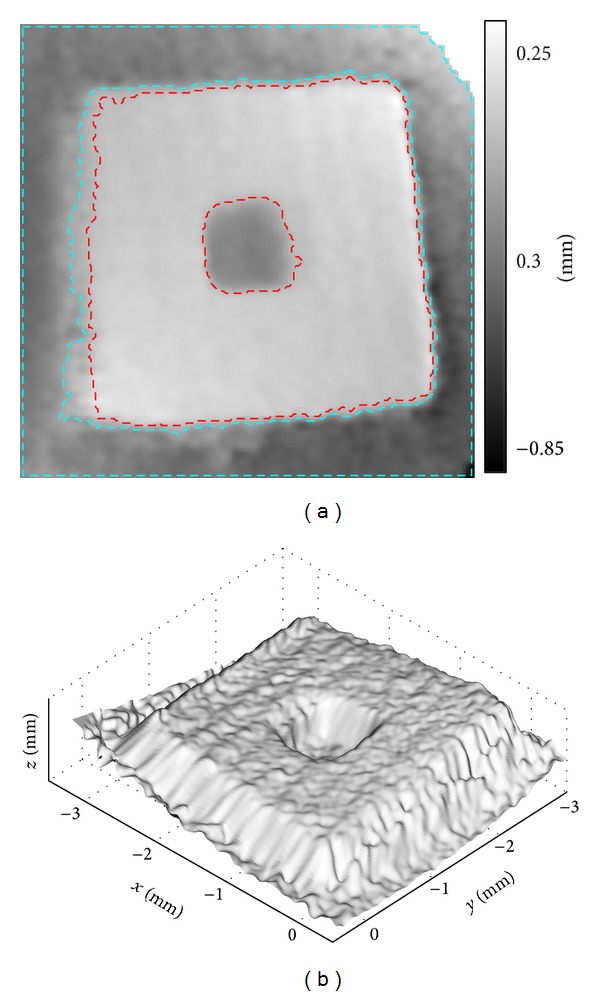
The gross tilt is removed from Figure 11.

The curvature is more obvious with the gross tilt removed by fitting a plane to the front surface and then subtracting the plane from the profile. The resultant 2D and 3D surface height distributions are shown in Figure 12. The distortion is most likely caused by the lens relay of the projection and imaging optics, which deviates from the pinhole model assumed in camera calibration and phase mapping. The deviation is small in the plane of the reference but is quite significant out of plane. Consequently, the results of the first experiment are relatively accurate but those of the second are poor. The average height difference of the front surface and the base plane, indicated in Figure 12(a), is 0.44 mm. Though it only differs from the result of the calliper by 20%, the deviation is quite severe because the surface is not even flat.

**Figure 13:**
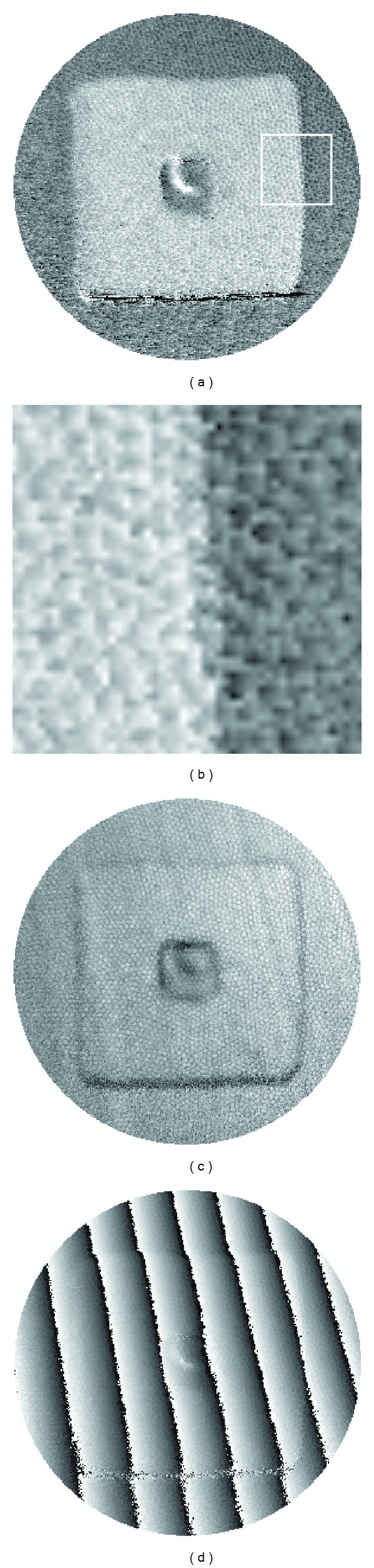
Underwater experiment. (a) Unfiltered height map. (b) Magnified white square region in (a). (c) Fringe contrast map. (d) Wrapped phase map.

Figure 13 shows the unfiltered results of the underwater experiment. In the 2D surface height map in Figure 13(a), strong speckle noise is observed. A region indicated by a white square is magnified in Figure 13(b), where the density and frequency of the noise are clearly visualized. As mentioned earlier, the noise is due to void regions of an imaging fiber. They are the spaces in between many individual fibers, as shown in the fringe contrast map in Figure 13(c). Bright spots in the contrast map are image of the individual fibers. They change their intensity according to the projection pattern, thereby gaining high contrast. The space in between individual fibers has lower contrast because no light passes through. Its intensity varies weakly, owing to the defocused light from the surrounding fibers. Phase errors are inevitable in these regions and have caused the significant amount of noise.

Another artifact in Figure 13(a) is the bright crescent region at the central dip. The root cause is quite unexpected; it is part of the image of the projector's bulb. In this experiment, constrained by the size of the vessel (see Figure 9(a)), the angle between the projection and imaging fibers is small; subsequently, the image of the bulb, produced by and beneath the water, is in the field of view. Without carefully arranging the two fibers, one may easily end up with a big bright spot (the image of the bulb) in the recorded images. We specifically put the imaging fiber closer to the specimen than the projection fiber so that the former blocked the spot light from the bulb. However, a residual edge of the spot light remains in the recorded fringe patterns; it has produced a low contrast region in Figure 13(c) and an erroneous phase distribution in the wrapped phase map; see Figure 13(d).

**Figure 14:**
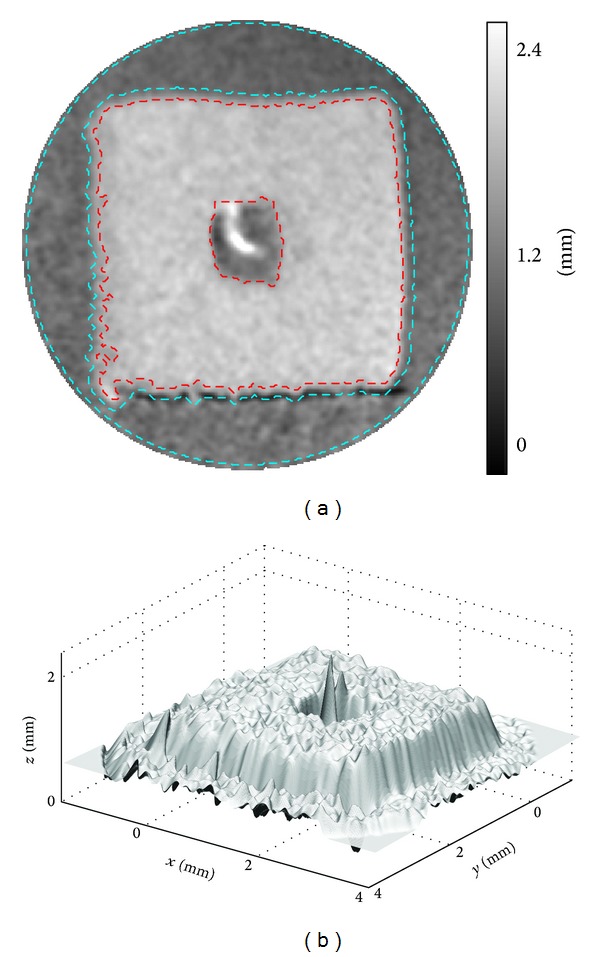
Underwater experiment. (a) Surface height map in 2D and (b) surface profile in 3D of the MEMS component.

The surface height map after smoothing exhibits improved uniformity but the artefact in the central region persists, as shown in Figure 14. The average height difference between the front surface and the base plane, indicated in Figure 14(a), is 0.65 mm. The relatively large deviation from 0.55 mm, obtained by the calliper, may be attributed to two factors. First, the angle between projection and imaging is small, leading to low sensitivity in height measurement. Second, the specimen was stuck to the base plane by double-faced tape, which might become less sticky in the water and the space between the two surfaces expanded slightly. Similar to the first experiment, the specimen was aligned perpendicular to the imaging fiber; hence, no curved distortion is present in the results.

Based on the three experiments, the unique feature of the fiber optic projection-imaging system is well demonstrated. However, it is also found that in using calibration and processing methods designed for conventional systems, noticeable error may be produced in scenarios where proper alignment is difficult. Furthermore, if the optics is in focus, which is considered necessary in common practice, an imaging fiber bundle will produce relatively strong fine-scale noise. The noise reduces the phase measurement accuracy and resolution but, to some extent, can be suppressed by smoothing filters.

## 5. Conclusion and Future Work

A fiber-based projection and imaging system is constructed for shape measurement. The experiments and results have demonstrated its strength and weakness. It is suitable for confined space applications and is able to reconstruct fairly accurate surface profile under proper alignment. When alignment is different, the system is able to retrieve the gross shape but is subject to noticeable distortion. Future work could focus on computational and instrumental approaches to mitigate the distortion. In the computational approach, a scaling factor can be incorporated in a modified camera model to take account of the second-order scale change with respect to different depth in *z* direction. (The first-order change is considered in the pinhole model.) In the instrumental approach, special couplers can be designed to tightly mount the projector, the bare lens, the objective lens, and the fiber bundle for projection. Such an integrated and fixed projection unit would require a once-only calibration, achieving the same level of compactness as an endoscope available on the market. A combination of the computational and instrumental approaches could reach a new generation of measurement devices, applicable to more complex objects. They can accommodate new applications difficult to embark previously and still maintain high measurement accuracy and flexibility of fringe projection.
